# COVID-19 and mucormycosis superinfection: Exploring the missing pathophysiological links

**DOI:** 10.1016/j.amsu.2021.102655

**Published:** 2021-08-06

**Authors:** Talal Almas, Waqas Nazar, Tarek Khedro, M. Ali Kanawati, Alishba Adnan, Mohammad Almuhaileej, Abdulaziz Alshamlan, Abdulaziz Abdulhadi, Kuvira T. Manamperi, Saba Sarfraz

**Affiliations:** RCSI University of Medicine and Health Sciences, Dublin, Ireland; Cavan General Hospital, County Cavan, Ireland; RCSI University of Medicine and Health Sciences, Dublin, Ireland; RCSI University of Medicine and Health Sciences, Dublin, Ireland; Karachi Medical and Dental College, Karachi, Pakistan; RCSI University of Medicine and Health Sciences, Dublin, Ireland; RCSI University of Medicine and Health Sciences, Dublin, Ireland; RCSI University of Medicine and Health Sciences, Dublin, Ireland; RCSI University of Medicine and Health Sciences, Dublin, Ireland; Islamabad Medical and Dental College, Islamabad, Pakistan

**Keywords:** COVID-19, Mucormycosis, Immunosupression, Mortality, Morbidity

## Abstract

The coronavirus disease 2019 continues to unearth new facets that portend grave clinical implications. In recent times, there has been mounting fervor regarding coronavirus disease 2019 and mucormycosis superinfection. While the correlation between the two is conspicuous, the underlying pathophysiological mechanisms that render a patient with coronavirus disease 2019 susceptible to mucormycosis, or vice versa, are still elusive.

## Highlights

• In recent times, there has been mounting fervor regarding coronavirus disease 2019 and mucormycosis superinfection.
• While the correlation between the two is conspicuous, the underlying pathophysiological mechanisms that render a patient with coronavirus disease 2019 susceptible to mucormycosis, or vice versa, are still elusive.
• Coupled with the urgent need to treat the cytokine storm via immunosuppressive steroids, the resultant host condition provides an optimal environment for the fungus to thrive.

## 1 Introduction

From its detrimental effects on various organs such as the liver to its potential long-term neurological effects, coronavirus-2019 (COVID-19) has continued to ravage healthcare systems across the world [1,2]. With the advent and distribution of vaccinations worldwide, at a historic pace, the world is slowly paving the way for a return to normalcy. However, the pandemic continues to reveal new challenges. One of these is superinfection with mucormycosis, an opportunistic fungal infection. Pre-pandemic, its prevalence has been primarily observed in immunocompromised patients, such as those with uncontrolled diabetes mellitus, neutropenia, hematological malignancies, and similar conditions [1]. Herein, we chronicle the relationship between COVID-19 and mucormycosis, collating the available sparse literature and positing future directions.

Untreated, the “black fungus” mucormycosis is rapidly fatal. Mucormycosis is caused by various fungi species from the Mucorales order [3]. As Mucorales spores exist widely in nature, it is possible for them to be present in the nasal mucosa of healthy people as a commensal organism [4]. If the patient develops a state of immunosuppression, however, this is when it may germinate pathologically within the paranasal sinuses and spread to nearby structures such as the orbit and, even worse, intracranially [5]. In Europe most cases have been identified with *Rhizopus* spp. (34 %), *Mucor* spp. (19 %), and *Lichtheimia* spp. (19 %) [3]. Its etiology, however, varies based on geography. For example, many cases in Australia were non-*Rhizopus* species infecting immunocompetent patients through trauma, yet these were limited to local infections. On the other hand, necrotizing fasciitis due to infection from intramuscular injections have been reported in India, and these cases were due to much rarer species of the Mucorales order.

The global incidence of mucormycosis has risen over the past few decades, yet these rising statistics have been virtually restricted to developing countries like India. In the United States and Europe, the prevalence is 0.01–0.02 per 100,000 population [3]. Meanwhile, India sees a prevalence of 14 per 100,000. In adults, the mortality rate ranges from 20 % to 100 % due to comorbidities, site of infection, treatment availability, and other factors such as quality healthcare systems. In children, the rate is 33.3 %. Historically, the rarity of mucormycosis has been the major obstacle in research [3]. This has prevented large studies and clinical trials that would typically elucidate information on its epidemiology, diagnosis, and treatment. It is treated with amphotericin-B, a last-line antifungal reserved for serious, systemic fungal infections. The only new antifungal drug with activity against Mucorales is isavuconazole; however, it offers little benefit over amphotericin-B and is replete with severe side effects.

## 2 Pathophysiology

COVID-19 has been shown to enter the cell via the ACE2 and TMPRSS-2 receptors. While ACE2-R is a ubiquitous receptor in the body, it has higher rates of expression in respiratory, renal, and gastrointestinal epithelium. TMPRSS-2 receptors are similar: they are present on many epithelia but especially on that of respiratory and gastrointestinal [1,2]. It has a propensity for attacking lymphocytes by binding to ACE2 receptors on these cells, inducing lymphopenia, reducing CD4^+^ and CD8^+^ T-cell counts and consequently reducing immunity levels. This damage is compounded with the raised interleukin levels (IL-2, IL-6, IL-7, interferon gamma inducible factor, granulocyte colony stimulating factor), effectively achieving a state of cytokine storm [4,6]. This causes atrophy of lymphoid tissue, weakening the defense system reserve pool, and preventing future production and proliferation of protective lymphocytes.

Furthermore, there is lactic acidosis, which destroys type II alveolar cells—the regenerative lung cells—leading to a plethora of respiratory difficulties that exacerbate acid-base levels. Eventually, this causes hypoxemia and hypoperfusion. Therefore, tissues depend on anaerobic metabolism that worsens the already present acidic conditions. Coupled with the urgent need to treat the cytokine storm via immunosuppressive steroids, all of this promotes an optimal environment for the fungus to thrive [7]. Finally, two other conditions fuel Mucorales growth in the infected body: raised ferritin levels due to increased hemolysis (iron is toxic to phagocytes) and a raised body temperature (they are thermotolerant organisms) [8]. The fungus receives its nutrition from ACE-2 mediated damage to pancreatic beta cells that results in and elevated plasma glucose levels [[9], [10]]. In fact, this explains why mucormycosis is more prevalent in those with diabetes: it thrives when there is an abundance of sugar. In a study in Mexico reviewing mucormycosis cases, diabetes was an underlying comorbidity in up to 72 % of patients [3]. As mucormycosis invades blood vessels via endothelial damage, the insulin resistance and raised glucose levels result in proliferation of the fungus and progressive weakening of an already shattered immune system. These catastrophic consequences can only mean the eventual deterioration of the patient. While Mucorales can be a commensal organism in immunocompetent patients, the severe cases of COVID-19 infection are often immunocompromised and are hospitalised for longer, with some even requiring long-term mechanical ventilation. This ventilation further makes them susceptible to infections like mucormycosis.

## 3 Literature review

We perused the PubMed, MEDLINE, and SCOPUS databases using the medical subject headings (MeSH) “Coronavirus disease 2019”, “COVID-19” AND “Mucormycosis.” Articles in languages other than English were excluded. Case reports, case series, correspondence articles, and editorials were included in the present review. A total of 12 cases were retrieved, comprising 11 males and 1 female. The mean age of onset was 48±17 years. Notably, the mortality rate hovered at a soaring 33.3 %, further invoking the notion of early intervention in afflicted patients. These outcomes are depicted in Table 1.

**Table 1:** A tabulation of the outcomes of literature review of patients co-infected with COVID-19 and mucormycosis to date.

Author	Age and Sex	Patient Characteristics	Radiological findings	Laboratory parameters	Treatment	Prognosis
Pasero et al.^4^	66 y/o Male	Multiple organ dysfunction, (SOFA)^a^	CT scan: Opacification of the left maxillary sinus and sclerosis. The **Thoracic scans**: cavitary lesions in the upper lobe of left lung.	Second BAS tes: cotton-candy colonies on (SDA)^b^	Liposomal Amphotericin B, 5 mg kg−1 IV.	Died
Johnson et al.^11^	79 y/o Male	Co-morbs: DM, HTN. Developed hypoxic respiratory failure.	CT chest: extensive bilateral pneumonia and new development of bilateral upper lobe cavitations	KOH preparation, culture, and isolation from the BAL culture tested positive for Mucormycosis infection.	Changed to IV L-AmB 400 mg daily for suspected mucormycosis infection.	Patient remained on ventilator support, tolerated IV L-AmB treatment, and was discharged to a long-term acute care facility
Krishna et al.^12^	22 y/o Male	Co-morbs: Hypothyroidism. Recurrent episodes of vasoplegic shock and multi-organ dysfunction.	CTPA^c^: segmental pulmonary embolism in the left lower lobe & peripheral consolidation in lower lobes and right upper	Autopsy findings: hematogenous dissemination, necrotic vasculitis, and cerebral invasion	Discovery of mucormycosis was post-mortem hence no treatment was opted.	Died due to cardiac tamponade
Veisi et al.^13^	40 y/o Female	Bilateral visual loss and complete ophthalmoplegia of the right eye	Orbital CT and MRI scans confirmed Mucormycosis presence.	Histopathological examination of paranasal sinuses showed granulomatous inflammation; positive Mucormycosis	Systemic amphotericin B and daily endoscopic sinus debridement and irrigation with diluted amphotericin B	She died because of dissemination into CNS
Veisi et al.^13^	54 y/o Male	Co-morbs: DM. Vision loss, proptosis, orbital inflammation, and complete ophthalmoplegia on the left side	CT scan revealed unilateral opacifications of the left orbit and paranasal sinuses	Necrotizing granulomatous inflammation with hyphae.	Systemic amphotericin B and daily endoscopic sinus debridement and irrigation with diluted amphotericin B	2 months later: discharged with oral posaconazole (800 mg/day)
Maini et al.^14^	38 y/o Male	Chemosis and pain in the left eye	Mucormycosis infection confirmed after MRI (polypoidal mucosal thickening involving left maxillary and ethmoid sinuses, anterior displacement of right eye) & FESS^d^	Histopathological findings: aseptate broad based hyphae grown on SDA and stained with lactofuchsin	IV Fluconazole & Amphotericin B, followed by surgical debridement	Recovered with minimal residual deformity
Baskar et al.^15^	28 y/o Male	Sudden vision loss and swelling of the right eyes.	CE CT^e^ scan of nose and paranasal sinus with orbit: right intraconal and retrobulbar soft tissue density along with mucosal thickening in the right ethmoid sinus	Tissue biopsy: branched aseptate hyphae confirming mucormycosis.	Liposomal amphotericin-B for rhino-orbital mucormycosis. Right-side orbital exenteration and ethmoid sinus debridement	Disease free at 2 month follow up.
Arana et al.^16^	62 y/o Male	Co-morbs: DM. fever, headache, and left malar region swelling	Facial CT: left maxillary sinusitis	Swab culture showed *Rhizopus oryzae*	Amphotericin B and an azole (initially isavuconazole and subsequently posaconazole. 6 surgical debridement procedures. Total left maxillectomy	Recovery after 5 months.
Arana et al.^16^	48 y/o Male	Co-morbs: Hx^f^ of CKD^g^. pain and an increase of lower right limb diameter	Unavailable	Culture from necrotic tissue showed *Lichtheimia ramosa* (musculoskeletal mucormycosis)	Liposomal amphotericin B 5 mg/kg q24h together with isavuconazole (3 months) 200 mg/8 h for 24 days	Recovered after 3 months
Sai Krishna et al.^17^	34 y/o Male	Co-morbs: HTN^h^ & DM. Continuous pain and swelling over the right side of the midface since 2 months.	CBCT^j^: aggressive osteolytic lesions in the right maxilla	Biopsy, curettage and saucerization: Fungal osteomyelitis.	IV liposomal Amphotericin B 5 mg/kg/day. Surgical resection via Weber Ferguson approach. Later antifungal drug was changed to oral Itraconazole 200 mg.	2 months; no signs of disease.
Sai Krishna et al.^17^	50 y/o Male	Co-morbs: uncontrolled DM. Swelling over the right side of the midface since 2 months	3D CT: mucormycosis of right maxilla and zygoma	Thick-walled aseptate fungal hyphae with right-angled branching in P.A.S. stain^i^: mucormycosis of the right maxilla	IV liposomal Amphotericin B 250 mg	Recovered 2 months later.
Bellanger et al.^18^	55 y/o Male	Feverish, with worsened respiratory function.	Chest CT: non-specific bilateral ground glass opacities suggesting COVID 19 infection.	Tracheal aspirate and BALF^k^ positive in culture for both *Aspergillus fumigatus* and *Rhizopus microsporus*.	Liposomal amphotericin B began at day 23 (5 mg/kg)	Died at day 40.

Abbreviations.

a Syndrome with sequential organ failure assessment (SOFA).

b Sabouraud dextrose agar (SDA).

c CTPA: CT pulmonary angiography.

d Functional Endoscopic Sinus Surgery (FESS).

e Contrast enhanced CT: CE CT.

f Hx: History.

g CKD: Chronic kidney disease.

h HTN: Hypertension.

i PAS stain: Periodic acid–Schiff–diastase stain.

j Cone-beam computed tomography systems (CBCT).

k Broncho-alveolar lavage fluid.

The studies in the table describe patient outcomes of a Mucormycosis infection in the context of COVID-19. The key takeaway is clear: mortality and morbidity are very high. COVID-19 complications have been a major focus and thus repeatedly described in numerous published studies since its initial outbreak; the potential havoc that a COVID-19 infection can wreak on the immune system thus lends a helping hand to the opportunistic Mucormycosis-causing bugs.

## 4 Conclusion

As there is currently no standard protocol that is being implemented to treat Mucormycosis in COVID-19 patients, there is undoubtedly an overwhelming need to formulate one. Moreover, the results of the current study have ramifications beyond a COVID-19 and Mucormycosis superinfection. While COVID-19 has been shown to produce an immunosuppressed state on its own, the need for a standard protocol is further necessitated when considering COVID-19 patients with concurrent immunosuppressive conditions, as they are even more susceptible to Mucormycosis and its deleterious effects and often fatal outcome. The optimal strategy would be to find an equilibrium between a testament regimen that does not overly suppress the immune system while also resolving the ongoing cytokines storm characteristic of severe COVID-19.

Given these evolving challenges, it is imperative that large scale trials are designed—not just to discern the interplay between COVID-19 and Mucormycosis, but also other immunosuppressive states that can portend significant morbidity and mortality.

## 5 Limitations

The article has some limitations. For instance, having a prior diagnosis of diabetes mellitus is a well-established predisposing factor for developing a mucormycosis infection. The causality between COVID-19 and mucormycosis cannot be reliably drawn. Nevertheless, there is a conspicuous correlation between the two that cannot be discounted. Finally, while Mucormycosis infection can be seen worldwide, it has a higher prevalence in the more underdeveloped regions of the world. Thus, published cases are much more likely to come from these regions.

## Provenance and peer review

Not commissioned, externally peer reviewed.

## Funding

None.

## Annals of medicine and surgery

The following information is required for submission. Please note that failure to respond to these questions/statements will mean your submission will be returned. If you have nothing to declare in any of these categories then this should be stated.

## Please state any conflicts of interest

NA

## Please state any sources of funding for your research

NA.

## Ethical approval

NA.

## Consent

NA.

## Author statement

TA, WN, TK: conceived the idea, designed the study, and drafted the manuscript.

MAK, AA, MA: conducted literature search and created the illustrations.

AA, AA, KTM, SS: revised the manuscript critically and refined the illustrations.

TA and WN: revised the final version of the manuscript critically and gave the final approval.

## Registration of research studies

1 Name of the registry: NA.
2 Unique Identifying number or registration ID: NA.
3 Hyperlink to your specific registration (must be publicly accessible and will be checked): NA.

## Guarantor

Talal Almas

## Declaration of competing interest

None.

## References

[1] Pandiar D., Kumar N.S., Anand R., Kamboj M., Narwal A., Shameena P.M. (2021 Jul). Does COVID 19 generate a milieu for propagation of mucormycosis?. Med. Hypotheses.

[2] Almas T., Ehtesham M., Khedro T., Haadi A., Nabeel A.R., Hussain S. (2021 Apr). The many faces of coronavirus disease 2019: COVID-19 and the liver. Ann Med Surg (Lond).

[3] Skiada A., Lass-Floerl C., Klimko N., Ibrahim A., Roilides E., Petrikkos G. (2018 Apr). Challenges in the diagnosis and treatment of mucormycosis. Med. Mycol..

[4] Pasero D., Sanna S., Liperi C., Piredda D., Branca G.P., Casadio L. (2020 Dec). A challenging complication following SARS-CoV-2 infection: a case of pulmonary mucormycosis. Infection.

[5] Sharma S., Grover M., Bhargava S., Samdani S., Kataria T. (2021 May). Post coronavirus disease mucormycosis: a deadly addition to the pandemic spectrum. J. Laryngol. Otol..

[6] Aggarwal S., Gollapudi S., Gupta S. (1999). Increased TNF-alpha-induced apoptosis in lymphocytes from aged humans: changes in TNF-alpha receptor expression and activation of caspases. J. Immunol..

[7] Fischer K., Hoffmann P., Voelkl S., Meidenbauer N., Ammer J., Edinger M. (2007 May). Inhibitory effect of tumor cell-derived lactic acid on human T cells. Blood.

[8] Wu C., Chen X., Cai Y. (2020). Risk factors associated with acute respiratory distress syndrome and death in patients with coronavirus disease 2019 pneumonia in wuhan, China. JAMA Intern Med.

[9] Yang J.K., Lin S.S., Ji X.J., Guo L.M. (2010). Binding of SARS coronavirus to its receptor damages islets and causes acute diabetes. Acta Diabetol..

[10] Yang X., Yu Y., Xu J., Shu H., Xia J., Liu H. (2020 May). Clinical course and outcomes of critically ill patients with SARS-CoV-2 pneumonia in Wuhan, China: a single-centered, retrospective, observational study. Lancet Respir Med.

[11] Johnson A.K., Ghazarian Z., Cendrowski K.D., Persichino J.G. (2021 Jun). Pulmonary aspergillosis and mucormycosis in a patient with COVID-19. Medical Mycology Case Reports.

[12] Krishna V., Morjaria J., Jalandari R., Omar F., Kaul S. (2021). Autoptic identification of disseminated mucormycosis in a young male presenting with cerebrovascular event, multi-organ dysfunction and COVID-19 infection. IDCases.

[13] Veisi A., Bagheri A., Eshaghi M., Rikhtehgar M.H., Rezaei Kanavi M., Farjad R. (2021 Apr). Rhino-orbital mucormycosis during steroid therapy in COVID-19 patients: a case report. Eur. J. Ophthalmol..

[14] Maini A., Tomar G., Khanna D., Kini Y., Mehta H., Bhagyasree V. (2021 May). Sino-orbital mucormycosis in a COVID-19 patient: a case report. Int J Surg Case Rep.

[15] Baskar H.C., Chandran A., Reddy C.S., Singh S. (2021 Jun). Rhino-orbital mucormycosis in a COVID-19 patient. BMJ Case Rep..

[16] Arana C., Cuevas Ramírez R.E., Xipell M., Casals J., Moreno A., Herrera S. (2021 May). Mucormycosis associated with COVID-19 in two kidney transplant patients. Transpl. Infect. Dis..

[17] Sai Krishna D., Raj H., Kurup P., Juneja M. (2021 May). Maxillofacial infections in covid-19 era-actuality or the unforeseen: 2 case reports. Indian J. Otolaryngol. Head Neck Surg..

[18] Bellanger A.P., Navellou J.C., Lepiller Q. (2021). Mixed mold infection with Aspergillus fumigatus and Rhizopus microsporus in a severe acute respiratory syndrome Coronavirus 2 (SARS-CoV-2) patient [published online ahead of print, 2021 Jan 27]. Infect Dis Now.

